# The Timing of Stroke Care Processes and Development of Stroke Associated Pneumonia: A National Registry Cohort Study

**DOI:** 10.3389/fneur.2022.875893

**Published:** 2022-04-13

**Authors:** Marco Antonio Lobo Chaves, Matthew Gittins, Benjamin Bray, Andy Vail, Craig J. Smith

**Affiliations:** ^1^Division of Cardiovascular Sciences, School of Medical Sciences, University of Manchester, Manchester, United Kingdom; ^2^Geoffrey Jefferson Brain Research Centre, Manchester, United Kingdom; ^3^Centre for Biostatistics, University of Manchester, Manchester, United Kingdom; ^4^School of Population Health and Environmental Sciences, King's College London, London, United Kingdom; ^5^Manchester Centre for Clinical Neurosciences, Geoffrey Jefferson Brain Research Centre, Manchester Academic Health Science Centre, Salford Royal National Health Service (NHS) Foundation Trust, Salford, United Kingdom

**Keywords:** stroke care, stroke complications, big data, stroke associated pneumonia, stroke care process

## Abstract

**Introduction:**

Timely stroke care can result in significant improvements in stroke recovery. However, little is known about how stroke care processes relate to complications such as the development of stroke associated pneumonia (SAP). Here we investigated associations between stroke care processes, their timing and development of SAP.

**Methods:**

We obtained patient-level data from the Sentinel Stroke National Audit Programme for all confirmed strokes between 1st April 2013 and 31st December 2018. SAP was identified if new antibiotic initiation for pneumonia occurred within the first 7 days of admission. Time to key stroke care processes in the pre-hospital, hyperacute and acute phase were investigated. A mixed effects logistic regression model estimated the association between SAP [Odds ratios (OR) with 95% CI] and each process of care after controlling for pre-determined confounders such as age, stroke severity and comorbidities.

**Results:**

SAP was identified in 8.5% of 413,133 patients in 169 stroke units. A long time to arrival at a stroke unit after symptom onset or time last seen well [OR (95% CI) = 1.29 (1.23–1.35)], from admission to assessment by a stroke specialist [1.10 (1.06–1.14)] and from admission to assessment by a physiotherapist [1.16 (1.12–1.21)] were all independently associated with SAP. Short door to needle times were associated with lower odds of SAP [0.90 (0.83–0.97)].

**Conclusion:**

Times from stroke onset and admission to certain key stroke care processes were associated with SAP. Understanding how timing of these care processes relate to SAP may enable development of preventive interventions to reduce antibiotic use and improve clinical outcomes.

## Introduction

Stroke Associated Pneumonia (SAP) occurs in around 7–13% of patients during the first 7 days of admission with a stroke (1). SAP is independently associated with worse functional outcomes, increased mortality and healthcare costs related to stroke care (2). Identification of potentially modifiable factors associated with SAP could ultimately lead to improved patient outcomes and reduce antibiotic use, which is a priority in an era of increasing antimicrobial resistance (3).

There are currently few strategies available to reduce the risk of SAP. Early swallow assessment and receipt of organized stroke unit care are associated with lower risk of SAP development (4, 5), yet little is known about how other care processes relate to risk of SAP. Preventive antibiotics have not been shown to reduce the risk of SAP or improve clinical outcomes. Other interventions, such as oral care and metoclopramide may reduce the risk of SAP, but require further evaluation (6, 7).

Better understanding the relationships between stroke care processes, their timing and the risk of SAP could provide a basis for interventions to improve patient outcomes and antibiotic stewardship. The main aim of this study was therefore to investigate the associations between the timing of stroke care processes spanning pre-hospital, hyperacute and acute care and the development of SAP.

## Methods

### Study Design

We undertook an observational cohort study using patient level data from the Sentinel Stroke National Audit Programme (SSNAP), for all confirmed and recorded strokes between the 1st of April 2013 and the 31st of December 2018 in England and Wales. Stroke units with fewer than 150 stroke admissions per year were excluded from the study, in order to focus on specialist stroke units and not on those repurposed for occasional patients (8).

### Data Source

SSNAP is a mandatory stroke registry implemented in 2013 by the Intercollegiate Stroke Working Party. It records baseline characteristics, demographic details and stroke care information in order to facilitate continued quality improvement of stroke care across England, Wales and Northern Ireland (9). The Healthcare Quality Improvement Partnership (HQIP) governs SSNAP, and made the final approval of the data transfer. Data access requests should be directed to HQIP.

### Clinical Characteristics

We extracted baseline patient characteristics, including the following vascular and SAP risk factors: age on admission, sex, total baseline National Institutes of Health Stroke Scale (NIHSS) score, pre-stroke modified Rankin Scale (mRS) score, diabetes, atrial fibrillation (AF), hypertension, congestive heart failure, previous stroke or transient ischaemic attack (TIA) and presence of dysphagia.

### Stroke Care Phases and Processes

As SAP occurs most often during the first 72 h of admission (1), we focused on stroke care processes during the pre-hospital, hyperacute and acute phases of the stroke pathway. A stroke care process was defined as a time to event variable, i.e., the time it took the patient from a specific event to receiving the specified care. We categorized time into quartiles to avoid distributional issues with outliers and allow interpretable incorporation of “unrecorded” and “not applicable” categories in our analysis. The selected stroke care processes included time from symptom onset or time last seen well to arrival at stroke unit, time from arrival at hospital to be assessed by a stroke nurse or to have a swallow assessment, time from arrival at hospital to receiving thrombolysis (door-to-needle time), time from arrival at hospital to be assessed by a stroke specialist doctor and time from arrival at hospital to be assessed by a physiotherapist (up to 72 h from admission). As swallow screens are generally performed by the stroke assessment nurse, there was high collinearity between the two processes. We created the aforementioned care process to address this collinearity. We selected these care processes after preliminary analysis of the data, discussions with the SSNAP team, consideration of data availability and discussions with a stroke specialist paramedic with experience of SSNAP data extraction and analysis. In all of our care process categories, with the exception of thrombolysis, the first quartile was selected as the reference. For thrombolysis, those patients who did not receive thrombolysis were selected as the reference category. We modeled thrombolysis differently, because the process of thrombolysis incorporates additional time-limited processes compared to patients that do not undergo thrombolysis, such as expedited CT scanning and assessments to facilitate optimal door-to-needle time. Patients who were discharged to another stroke center were dropped from the analysis to avoid potential double inclusion within the study.

### Statistical Analysis and Data Structure

The primary outcome measure was SAP defined here within SSNAP as new antibiotic initiation for suspected pneumonia started within the first 7 days of admission. The data had a hierarchical structure with stroke patients clustered within stroke units. A mixed-effects logistic regression modeled the binary presence of SAP vs. no-SAP. The care processes for both SAP and non-SAP patients were described using standard descriptive statistics. Clinical characteristics and stroke care processes were modeled as fixed effects with stroke unit modeled as the random intercept. We modeled each care process individually with the clinical characteristics and then modeled each care process based on VanderWeele's principals of confounder selection to avoid adjusting for factors thought to be on the causal pathway (10). A sensitivity analysis was performed for all patients whose length of stay was 7 days or more to ensure there was no misclassification due to the time component associated with the definition of SAP in our study. Odds ratios (ORs) and 95% confidence intervals (CIs) are reported. All statistical analysis was performed in Stata v.14 IC.

## Results

The dataset comprised 456,590 patients across 322 units. After excluding the units with fewer than 150 stroke admissions per year, the final dataset comprised 413,133 patients across 169 stroke units. SAP was recorded in 34,987 (8.5%) patients. A full description of the clinical characteristics is presented in Supplementary Table S1. The median time from symptom onset to arrival at a stroke unit was 7.35 h (IQR 4.20–20 h). A total of 47,240 patients (11.4%) received thrombolysis, comprising 42,117 (89.2%) non-SAP patients and 4,618 (9.8%) SAP patients. The median time from arrival at hospital to be seen by a stroke specialist doctor was 11.6 h (IQR 2.0–20.3 h). A full description of the times and cut-offs based on quartiles can be found in Supplementary Table S2.

**Figure 1:**
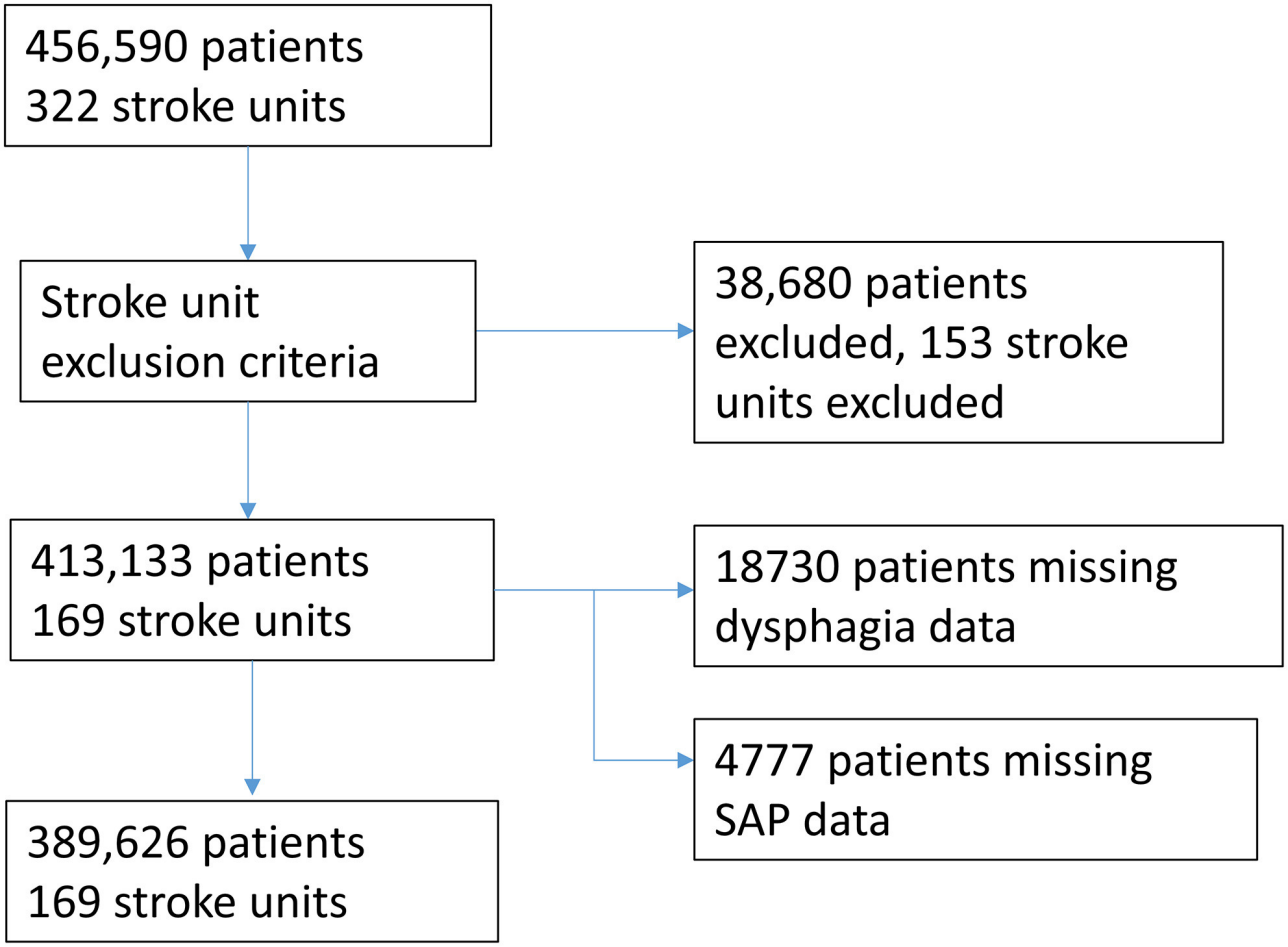
Flow diagram showing exclusion criteria and missing data to arrival at final sample size.

The analyzed sample comprised of 389,626 patients across 169 stroke units after also accounting for missing data for SAP and dysphagia (Figure 1).

For pre-hospital care, the patients who arrived at the stroke unit within the third and fourth quartiles was independently associated with SAP (OR of 1.1, 95% CI 1.0–1.1 and OR of 1.2, 95% CI 1.2–1.3, respectively) (Table 1). Patients with door to needle time in the first and second quartile were associated with decreased odds of SAP, with an OR of 0.89 (95% CI 0.82–0.96) in the first quartile and an OR of 0.94 (95% CI 0.87–1.0) in the second quartile. Patients who were assessed by a stroke specialist doctor in the fourth quartile had an increased odds of SAP with an OR of 1.1 (95% CI 1.0–1.1). Patients who were assessed by a physiotherapist in the fourth quartile were associated with increased odds of SAP with an OR of 1.2 (95% CI 1.1–1.2).

**Table 1:** Multivariable multilevel logistic regression odds ratios for stroke care processes describing their association with SAP.

**Stroke care process**	**Odds ratio (individual care process)**	**95% confidence interval**	**Odds ratio (VanderWeele confounder selection)**	**95% Confidence interval**
**Time from symptom onset to arrival at stroke unit**				
1st quartile (<4.20 h)	1.0 (reference)	–	1.0 (reference)	–
2nd Quartile (4.20–7.35 h)	1.06	1.02–1.10	1.06	1.0–1.10
3rd Quartile (7.35–20 h)	1.12	1.07–1.16	1.12	1.07–1.16
4th Quartile (>20 h)	1.29	1.23–1.35	1.29	1.23–1.35
Unknown	1.14	1.10–1.18	1.14	1.10–1.18
**Time from arrival at hospital to be assessed by a stroke nurse or have a swallow screen (composite)**				
1st quartile (<10 min)	1.0	–	1.0 (reference)	–
2nd quartile (10–90 min)	0.96	0.92–1.0	0.96	0.92–1.01
3rd quartile (90–260 min)	0.97	0.93–1.0	0.97	0.92–1.02
4th quartile (>260 min)	1.0	0.96–1.1	0.99	0.93–1.03
Unknown	1.1	1.0–1.1	1.04	0.99–1.10
**Door to needle time**				
Did not receive thrombolysis	1.0 (reference)	–	1.0 (reference)	–
1st quartile (<40 min)	0.83	0.76–0.89	0.90	0.83–0.97
2nd quartile (40–50 min)	0.87	0.82–0.94	0.95	0.88–1.02
3rd quartile (50–80 min)	0.94	0.88–1.0	1.02	0.95–1.10
4th quartile (>80 min)	1.0	0.94–1.1	1.06	0.99–1.14
**Time from arrival to be assessed by a stroke specialist doctor**				
1st quartile (<2 h)	1.0	–	1.0 (reference)	–
2nd quartile (2–11.6 h)	1.1	1.0–1.1	1.02	0.99–1.06
3rd quartile (11.6–20.3 h)	1.1	1.0–1.1	1.01	0.97–1.05
4th quartile (>20.3 h)	1.2	1.1–1.2	1.10	1.06–1.14
Unknown	1.0	0.96–1.1	0.91	0.86–0.96
**Time from arrival to be assessed by a physiotherapist**				
1st quartile (<15.6 h)	1.0 (reference)		1.0 (reference)	–
2nd quartile (15.6–21.3 h)	0.97	0.94–1.01	0.96	0.93–1.00
3rd quartile (21.3–27.8 h)	1.0	0.97–1.04	0.98	0.95–1.02
4th quartile (>27.8 h)	1.2	1.2–1.3	1.16	1.12–1.21
Unknown	0.83	0.79–0.86	0.79	0.75–0.82

*VanderWeele confounder selection for each care process (clinical characteristics were selected in all care processes) −1. Arrival at stroke unit—clinical characteristics/2. Assessment by a stroke nurse or swallow screen—arrival to stroke unit/3. Door to needle time—arrival at stroke unit/4. Assessment by stroke specialist doctor—arrival at stroke unit and assessment by a stroke nurse or swallow screen/5. Assessment by physiotherapist—arrival at stroke unit, assessment by a stroke nurse or swallow screen and assessment by a stroke specialist doctor*.

In the sensitivity analysis, 186,633 (45.2%) patients who were discharged or died within the first 7 days were excluded across the dataset, leaving 231,277 patients, and 33,076 (14.3%) patients with SAP. Our findings were comparable to our main analysis, indicating that SAP was minimally misclassified. A full description of the sensitivity analysis can be found in Supplementary Table S3.

## Discussion

We found that those patients with longer times to arrival at a stroke unit, assessment by a stroke specialist doctor and assessment by a physiotherapist were independently associated with up to 30% increased odds of SAP for those above the median on each of these care process measures. We also found that shorter time to thrombolysis was associated with reduced odds of SAP. Whilst we cannot conclude causal relationships, there are plausible mechanisms that warrant exploration. These findings are of interest as they highlight components of the pre-hospital, hyperacute and acute care pathway, and delays in receiving them, could be important targets for interventions to reduce the risk of SAP.

Increased times to arrival at a stroke unit were associated with increased odds of SAP, independent of the other care processes and baseline characteristics such as age and stroke severity. Whilst we cannot exclude that shorter time to arrival at a stroke unit was a marker of more rapid stroke care after arrival, there could be several other potential explanations. Faster dispatch times could reduce the likelihood of aspiration, by reducing the “the time down” and by expediting interventions such as positioning (11, 12) and thrombolysis or thrombectomy. Conversely, we cannot exclude that some patients with later dispatch times already had evolving pneumonia which manifest clinically after arrival in hospital (13).

Previous studies have found that thrombolysis reduces the risk of SAP and that SAP is associated with worse outcomes in thrombolysed patients (14). We additionally found that door to needle time within 40 min was independently associated with reduced odds of SAP. However, there appears to be no difference in development of incident pneumonia between placebo and tissue plasminogen activator treated patients in randomized trials (15, 16). Our findings in real-world practice could be explained by early improvement in stroke severity (including level of alertness and dysphagia) in those with faster door to needle times. Another possible explanation is that immunosuppression induced by thrombolysis with tissue plasminogen activator, might potentially increase the susceptibility to SAP with treatment later rather than earlier in the thrombolysis time window (17, 18).

Delays in physiotherapy assessment and delays to assessment by a stroke specialist doctor were also associated with increased odds of SAP, independent of each other and the other care processes evaluated. As many patients develop SAP within the first 24 h (1), later assessment by the stroke specialist doctor could be a marker for late diagnosis of SAP and decision-making on antibiotic initiation. Another factor is that a stroke specialist would influence various other aspects of hyperacute and acute management such as thrombolysis or thrombectomy which could affect the odds of developing SAP. Physiotherapy interventions such as turning and mobilization and chest physiotherapy may reduce the risk of pneumonia in stroke (19, 20). However, we did not have access to any data detailing specific physiotherapy interventions in individual patients. Shorter times to assessment by a physiotherapist could facilitate earlier mobilization and positioning although there appears to be no data to support this from randomized trials (21, 22).

We cannot exclude the possibility of reverse causality in our findings. For example, patients with evolving or diagnosed with SAP could be more unwell, leading to delays in certain assessments e.g., by physiotherapy, or requiring longer at-scene assessments by the paramedics, leading to delayed or longer transfers to the stroke unit (2).

Our results needs to be interpreted with caution. Firstly, the inclusion of unknown times in each stroke care process has implications for interpretation. The unknown time to event for symptom onset to arrival at the stroke unit, time to be assessed by a stroke nurse, time to be assessed by a stroke specialist doctor, and time to be assessed by a physiotherapist indicated statistically significant results. This means interpretation of the other timing categories, as the relationship reported between time to event metrics and outcome could be biased if unknown time is not randomly distributed. Unknown time to arrival at a stroke unit could be associated with SAP if the patient experienced a milder stroke, delaying access to hospital, being diagnosed, or simply causing an arrival without a clear time of onset. This uncertainty could then impact upon time to be assessed by a stroke nurse. The decreased odds of SAP in the unknown time to be seen by a physiotherapist needs to be considered separately. Time to be seen by a physiotherapist is measured in SSNAP only if they were seen within 72 h from admission. If a patient was discharged, due to good health or death, before being seen by a physiotherapist, it could provide an explanation as to our results.

Our findings, if confirmed, would strengthen existing evidence for good quality and timely stroke care. Previous studies have suggested that timing of dysphagia assessment is also associated with development of SAP (5, 23). Considering how the timings of certain care processes in the pre-hospital, hyperacute and acute phase relate to SAP could lead to the development of interventions that can reduce the incidence of SAP and implications for antibiotic stewardship. Variation in the timings of care processes could also be a modifiable contributor to the observed variation in SAP frequency (24) and therefore merits further research.

Our study has several strengths and limitations. The SSNAP data contains unselected patient data with a high case-ascertainment providing generalizable findings that are representative of real-world events. Our observations for care process timing are likely to be relevant to stroke care beyond the UK. However, the lack of complete data for each of the stroke care processes across our dataset is a limiting factor. The data may be missing for reasons related to prognosis, which limits the conclusions that can be drawn from our study. In addition, the categorization of these continuous measures of time might increase the presence residual confounding. Another important limitation to highlight is the ambiguity associated with the definition of SAP used by SSNAP, which could lead to misleading conclusions. We were not able to include other potentially important stroke care processes that could be associated with SAP, such as time to CT scan, the amount of physiotherapy time each patient received or if the patient was intubated or not. While some these data are available to extract from SSNAP, we did not have access to this at the time of this study. It is also important to highlight that there are unmeasured aspects of stroke care which could provide further insight. Among these include the groin puncture time which could be playing an important role in the care process of thrombolysis. Finally, we were not able to account for organizational level factors, such as staffing levels, stroke unit size or geography. Organizational level factors may have been important confounders as they may represent directly or indirectly measures for the quality of care the stroke patients received.

## Conclusion

Increased time from symptom onset to arrival, thrombolysis door to needle time, time to be seen by a physiotherapist and time to be seen by a stroke specialist doctor were associated with increased odds of SAP even once measures of patient, stroke, and prior care process characteristics had been adjusted. Better understanding how the timing of these care processes relate to SAP may enable development of preventive interventions to reduce antibiotic use and improve clinical outcomes.

## Data Availability Statement

The data analyzed in this study was obtained from the Sentinel Stroke National Audit Programme (SSNAP), the following licenses/restrictions apply: A data request form(s) must be completed and submitted to SSNAP. If accepted, a Data Sharing Agreement and Data Access Request form will need to be completed and submitted for consideration by the Healthcare Quality Improvement Programme (HQIP). Requests to access these datasets should be directed to SSNAP, https://www.strokeaudit.org/Research/Data-requests.aspx.

## Ethics Statement

Ethical review and approval was not required for the study on human participants in accordance with the local legislation and institutional requirements. Written informed consent for participation was not required for this study in accordance with the national legislation and the institutional requirements.

## Author Contributions

ML submitted the data request, contributed to the data analysis, and the first draft. CS, AV, and BB designed the study. MG contributed with the data analysis. All authors contributed to the editing of the final draft. All authors contributed to the article and approved the submitted version.

## Conflict of Interest

The authors declare that the research was conducted in the absence of any commercial or financial relationships that could be construed as a potential conflict of interest.

## Publisher's Note

All claims expressed in this article are solely those of the authors and do not necessarily represent those of their affiliated organizations, or those of the publisher, the editors and the reviewers. Any product that may be evaluated in this article, or claim that may be made by its manufacturer, is not guaranteed or endorsed by the publisher.
