# Supplementary material for: The Timing of Stroke Care Processes and Development of Stroke Associated Pneumonia: A National Registry Cohort Study
Source: Front Neurol. 2022 Apr 13;13:875893. doi: 10.3389/fneur.2022.875893 (PMC9043446; doi:10.3389/fneur.2022.875893)
Supplement: Supplementary file 1 [file Table_1.docx]

| Clinical characteristic | Non-SAP | SAP | missing |
| --- | --- | --- | --- |
| *Female* | 184734 (90.0%) | 17980 (8.76%) | 2524 (1.22%) |
| *Age on arrival (y)* |  |  |  |
| <60 | 58604 (95.9%) | 1846 (3.02%) | 687 (1.12%) |
| 60-69 | 63839 (93.9%) | 3395 (4.99%) | 741 (1.09%) |
| 70-79 | 102229 (91.4%) | 8397 (7.51%) | 1182 (1.05%) |
| 80-89 | 114011 (87.5%) | 14646 (11.2%) | 1571 (1.20%) |
| >90 | 39463 (84.3%) | 6713 (14.3%) | 596 (1.27%) |
| *Ethnicity* |  |  |  |
| White | 337526 (90.4%) | 31889 (8.53%) | 4118 (1.10%) |
| Asian | 10919 (92.1%) | 756 (6.38%) | 177 (1.49%) |
| Black | 4617 (93.7%) | 252 (5.11%) | 60 (1.21%) |
| Mixed | 1272 (90.5%) | 110 (7.82%) | 24 (1.71%) |
| Other | 23812 (90.9%) | 1980 (7.56%) | 398 (1.52%) |
| *CHF* | 19022 (85.2%) | 2986 (13.4%) | 306 (1.37%) |
| *Hypertension* | 202701 (90.3%) | 19232 (8.57%) | 2432 (1.08%) |
| *Diabetes* | 78158 (90.4%) | 7390 (8.54%) | 933 (1.07%) |
| *Atrial fibrillation* | 71285 (86.0%) | 10501 (12.7%) | 1056 (1.27%) |
| *Previous stroke or TIA* | 99885(89.9%) | 10029 (9.03%) | 1196 (1.08%) |
| *Stroke subtype* |  |  |  |
| Ischaemic stroke | 333119 (90.9%) | 29460 (8.04%) | 3863 (1.05%) |
| ICH | 42272 (87.3%) | 5321(11.0%) | 814 (1.68%) |
| *mRS before stroke* |  |  |  |
| 0 | 212719 (93.1%) | 13156 (5.76%) | 2524 (1.11%) |
| 1 | 57772 (90.4%) | 5439 (8.51%) | 680 (1.06%) |
| 2 | 38017 (87.9%) | 4765 (11.0%) | 485 (1.12%) |
| 3 | 41547 (85.4%) | 6531 (13.4%) | 581 (1.20%) |
| 4 | 21678 (83.6%) | 3898 (15.0%) | 360 (1.39%) |
| 5 | 6413 (82.7%) | 1198 (15.4%) | 147 (1.89%) |
| *NIHSS on arrival (median, IQR)* | 4 (2 -9) n=330710 | 14 (6 - 20) n= 27800 | - |
| *Dysphagic* | 14394 (32.4%) | 27405 (61.7%) | 2614 (5.89%) |

**Table S1.** Summary data of clinical characteristics. TIA – Transient Ischaemic Attack, mRS – modified Rankin scale, ICH – Intracerebral haemorrhage
