# Supplementary material for: The Timing of Stroke Care Processes and Development of Stroke Associated Pneumonia: A National Registry Cohort Study
Source: Front Neurol. 2022 Apr 13;13:875893. doi: 10.3389/fneur.2022.875893 (PMC9043446; doi:10.3389/fneur.2022.875893)
Supplement: Supplementary file 2 [file Table_2.docx]

| Stroke care process | Non-sap | sap | missing |
| --- | --- | --- | --- |
| *Time from symptom onset to arrival at stroke unit* |  |  |  |
| 1^st^ quartile (<4.20 hrs) | 61033 (89.7%) | 6274 (9.22%) | 768 (1.12%) |
| 2^nd^ Quartile (4.20-7.35 hrs) | 61603 (90.0%) | 6157 (9.00%) | 716 (1.04%) |
| 3^rd^ quartile (7.35-20 hrs) | 62382 (91.4%) | 5239 (7.68%) | 599 (0.88%) |
| 4^th^ quartile >20 hrs | 62632 (91.4%) | 5277 (7.70%) | 588 (0.86%) |
| unknown time | 130496 (90.2%) | 12040 (8.32%) | 2106 (1.46%) |
| *did the patient receive thrombolysis* |  |  |  |
| No | 4418 (87.7%) | 422 (8.37%) | 200 (4.00%) |
| no but* | 331611 (90.8%) | 29947 (8.20%) | 4072 (1.11%) |
| yes | 42117 (89.2%) | 4618 (9.78%) | 505 (1.01%) |
| *Door to needle time* |  |  |  |
| 1^st^ quartile (<40 min) | 9732 (90.5%) | 943 (8.77%) | 74 (0.69%) |
| 2^nd^ quartile (40-50 min) | 9694 (89.4%) | 1048 (9.67%) | 98 (0.90%) |
| 3^rd^ quartile (50-80 min) | 10026 (88.7%) | 1139 (10.0%) | 134 (1.28%) |
| 4^th^ quartile (>80 min) | 9858 (88.7%) | 1098 (9.87%) | 164 (1.57%) |
| *time from arrival at hospital to be assessed by a stroke nurse or had a swallow screen (Composite)* |  |  |  |
| 1^st^ quartile (<10 min) | 30553 (90.1%) | 2993 (8.82%) | 373 (1.10%) |
| 2^nd^ quartile (10-90 min) | 76567 (91.3%) | 6550 (7.81%) | 721 (0.86%) |
| 3^rd^ quartile (90 – 260 min) | 63345 (91.6%) | 5289 (7.64%) | 551 (0.79%) |
| 4^th^ quartile (>260 min) | 73827 (92.0%) | 5700 (7.10%) | 711 (0.89%) |
| unknown time | 133854 (88.8%) | 14455 (9.60%) | 2415 (1.60%) |
| *Time from arrival to be assessed by a stroke specialist doctor* |  |  |  |
| 1^st^ quartile (<2hrs) | 87457 (90.2%) | 8563 (8.83%) | 959 (0.99%) |
| 2^nd^ quartile (2-11.6 hrs) | 88546 (90.7%) | 8130 (8.32%) | 952 (0.98%) |
| 3^rd^ quartile (11.6 – 20.3 hrs) | 89125 (91.6%) | 7125 (7.32%) | 984 (1.01%) |
| 4^th^ quartile (>20.3 hrs) | 87979 (90.3%) | 8438 (8.67%) | 1017 (1.04%) |
| unknown time | 25021 (87.4%) | 2731 (9.54%) | 865 (3.02%) |
| *time from arrival to be assessed by a physiotherapist* |  |  |  |
| 1^st^ quartile (<15.6 hrs) | 79006 (90.9%) | 7171 (8.25%) | 771 (0.89%) |
| 2^nd^ quartile (15.6 – 21.3 hrs) | 79951 (92.0%) | 6173 (7.11%) | 754 (0.87%) |
| 3^rd^ quartile (21.3 – 27.8 hrs) | 79502 (91.2%) | 6879 (7.07%) | 808 (0.93%) |
| 4^th^ quartile (>27.8hrs) | 78015 (89.6%) | 8266 (9.50%) | 818 (0.94%) |
| unknown time | 61672 (88.4%) | 6498 (9.31%) | 1626 (2.33%) |

**Table S2**. Summary data of stroke care processes and criteria used in SSNAP and included in the model. *No but category means the patient did not received thrombolysis due one of the following reasons: the patient arrived outside the thrombolysis window, associated co-morbidity, use of contraindicated medication, refusal of thrombolysis by the patient, age, the symptoms were improving, stroke either too mild or too severe, symptom onset time was unknown or for some other medical reason
