# Supplementary material for: The Timing of Stroke Care Processes and Development of Stroke Associated Pneumonia: A National Registry Cohort Study
Source: Front Neurol. 2022 Apr 13;13:875893. doi: 10.3389/fneur.2022.875893 (PMC9043446; doi:10.3389/fneur.2022.875893)
Supplement: Supplementary file 3 [file Table_3.docx]

| Stroke care phase/ clinical characteristic | Odds ratio | 95% Confidence Interval |
| --- | --- | --- |

| *Time from symptom onset to arrival at stroke unit* |  |  |
| --- | --- | --- |
| 1^st^ quartile | 1.0 (reference) | - |
| 2^nd^ quartile | 1.03 | 0.99 to 1.08 |
| 3^rd^ quartile | 1.07 | 1.02 to 1.12 |
| 4^th^ quartile | 1.17 | 1.12 to 1.23 |
| Unknown | 1.06 | 1.02 to 1.09 |
| *Time from arrival at hospital to be seen by a stroke nurse or have a swallow screen* |  |  |
| 1^st^ quartile | 1.0 (reference) | - |
| 2^nd^ quartile | 0.96 | 0.91 to 1.01 |
| 3^rd^ quartile | 0.97 | 0.92 to 1.02 |
| 4^th^ quartile | 0.97 | 0.92 to 1.03 |
| Unknown | 0.94 | 0.90 to 1.00 |
| *Time from arrival at hospital to thrombolysis* |  |  |
| Did not receive thrombolysis | 1.0 (reference) | - |
| 1^st^ quartile | 1.00 | 0.92 to 1.09 |
| 2^nd^ quartile | 1.02 | 0.95 to 1.11 |
| 3^rd^ quartile | 1.10 | 1.02 to 1.18 |
| 4^th^ quartile | 1.14 | 1.06 to 1.23 |
| *Time from arrival to be seen by a stroke specialist doctor* |  |  |
| 1^st^ quartile | 1.0 (reference) | - |
| 2^nd^ quartile | 1.03 | 0.99 to 1.07 |
| 3^rd^ quartile | 1.04 | 1.00 to 1.09 |
| 4^th^ quartile | 1.08 | 1.04 to 1.13 |
| Unknown | 0.81 | 0.77 to 0.86 |
| *Time from arrival to be seen by a physiotherapist* |  |  |
| 1^st^ quartile | 1.0 (reference) | - |
| 2^nd^ quartile | 0.98 | 0.93 to 1.02 |
| 3^rd^ quartile | 0.98 | 0.94 to 1.02 |
| 4^th^ quartile | 1.04 | 1.0 to 1.08 |
| Unknown | 0.71 | 0.67 to 0.74 |

**Table S3**. Sensitivity analysis regression results for stroke care processes. Care processes were included according to the same VanderWeele’s confounder selection described in table in main text
